# Effects of continuous cropping Jiashi muskmelon on rhizosphere microbial community

**DOI:** 10.3389/fmicb.2022.1086334

**Published:** 2023-01-09

**Authors:** Jilian Wang, Mingyuan Li, Qian Zhou, Tian Zhang

**Affiliations:** ^1^Department of Biologic and Geographic Sciences, Kashi University, Kashi, China; ^2^Key Laboratory of Biological Resources and Ecology of Pamirs Plateau in Xinjiang Uygur Autonomous Region, Kashi, China

**Keywords:** continuous cropping, Jiashi muskmelon, high-throughput absolute quantification sequencing, rhizosphere soil, bacterial community structure

## Abstract

**Introduction:**

The continuous cropping of crops can result in the deterioration of the soil environment and cause a decline in plant health and yield, which complicates agricultural production. However, the effects of continuous melon cropping on rhizospheric microbial communities remain poorly understood.

**Methods:**

In this study, high-throughput absolute quantification 16S rRNA gene amplicon sequencing was employed to analyze the bacterial community structure of greenhouse rhizosphere soil from Jiashi muskmelon replanted for 0, 1, 2, and 3 years (CK, 1a, 2a, and 3a, respectively).

**Results:**

The results showed that long- term continuous cropping caused significant changes in soil physicochemical properties. The bacterial absolute abundances increased, but the bacterial community richness and diversity were significantly lost (p < 0.05). The composition of bacterial community was more similar after 2 and 3 years of continuous cropping. The longer the continuous cropping years were, the greater the shift in the bacterial diversity and abundance. Analysis of potential functional components revealed that different bacterial groups were enriched in different continuous cropping years. The significant reduction of the taxa associated with nitrate reduction may be responsible for the loss of soil nitrogen in continuous cropping soil.

**Discussion:**

In summary, continuous cropping had a significant impact on the bacterial community structure of Jiashi muskmelon rhizospheric soil, and these results will provide a reference for soil management and scientific fertilization of melon and other crops under a continuous cropping regime.

## Introduction

Melon (*Cucumis melo*), with high nutritional value and medicinal value, is a popular fresh fruit loved by people all over the world. It is an important industrial crop that is widely cultivated in temperate and tropical climates, and China is the main producing area. In particular, Jiashi muskmelon is a kind of crop with good economic benefits and excellent quality in northwest China and has been a protected geographical indication product. To meet people’s demand for Jiashi muskmelon, large-scale intensive production has accelerated, and the area used for continuous cropping is increasing due to the shortage of arable land in China. The agronomic management costs were lower than the costs of rotation cropping (Reim et al., 2019), but continuous cropping soil sickness is common with crops (Wei et al., 2018; Chen et al., 2020; Wang W. H. et al., 2020; Chen et al., 2022). Long-term continuous cropping usually leads to many problems, such as deterioration of soil texture, increased crop disease, and reduced yields, which seriously affect the sustainable development of the melon industry. Farmers are trying to ease soil sickness by applying more fertilizers and pesticides, but this will lead to increased costs and excessive pesticide residues in crops, which threatens the health of consumers. In addition to forced fallow, reasonable rotation is still an effective way to respond to soil sickness (Henry et al., 2019; Kayikcioglu et al., 2020; Singh and Kumar, 2021; Zhang et al., 2021). However, for facility agriculture, crop rotation not only deprives the profit space of farmers but also increases the risk of investment.

Soil microorganisms are important parts of soil ecosystems and are involved in nutrient transformation, soil structure improvement, and harmful substance degradation (De Corato, 2020; Mącik et al., 2020). They also have a profound impact on soil-borne diseases (Lehmann et al., 2020). With the development of high-throughput sequencing technology, the interaction between soil microorganisms and continuous cropping practices has become a research hotspot (Liu X. et al., 2020; Yao et al., 2020; Li et al., 2021). The rhizosphere is the interface where plants, soil, and the microbiome frequently exchange materials and transmit signals. The interaction between plants, soil, microbial activity, and the environment forms a tight network. The network plays a critical role in defending against pathogen attacks, forming rhizosphere immunity. However, according to some reported studies, continuous cropping affects the rhizospheric microbial structure (Song et al., 2018; Pervaiz et al., 2020; Wang Y. et al., 2020). In turn, such alteration further contributes to the aggravation of continuous cropping soil sickness (Maguire et al., 2020).

Bacteria is the largest microorganism species in soil. They play important roles in decomposition of organic matter, humus synthesis, carbon and nitrogen cycle, and soil development. After continuous cropping, some plant pathogens were enriched, whereas microorganisms that can resist soil borne diseases and maintain plant health, such as *Pseudomonas* and *Bacillus*, showed an inhibitory impact (Li et al., 2016; Huang et al., 2019; Luo et al., 2019; Wang Q. X. et al., 2020). In addition, the number of functional bacteria involved in nutrient element cycling and the decomposition of harmful allopathic substances also showed negative feedback over time (Liu et al., 2021). Continuous cropping clearly has a long-term effect on the soil microbial community, and it may take decades to restore the original microbial community system (Bao et al., 2020). However, each continuous cropping system had different action mechanisms, especially in the composition of microbial community. To date, knowledge regarding the response of the rhizospheric bacterial community and functional groups to the continuous cropping of melon is still poorly understood.

Based on high-throughput sequencing technology, the understanding of bacterial communities in different ecological systems has been dramatically expanded, but it is still difficult to obtain an accurate population density. Absolute quantification 16S rRNA gene amplicon sequencing is a novel technology combining high-throughput 16S amplicon sequencing with absolute quantitative PCR (qPCR), which quantifies the species abundance by adding synthetic spike-in standard sequences to the sample DNA pool. It has the potential to clarify many pending questions in micro ecology (Tkacz et al., 2018; Jiang et al., 2019). This research describes a comparative analysis of the rhizosphere bacterial community of Jiashi muskmelon greenhouse soils with different cultivation histories by absolute quantification 16S rRNA gene amplicon sequencing. Based on FAPROTAX functional prediction, the potential functional components were also elucidated. The results obtained here will provide guidance for soil improvement and scientific fertilization of melon replanting.

## Materials and methods

### Study region and experimental description

The study field was located in Jiashi County (77°17′E, 39°41′N) of the Xinjiang Uygur Autonomous Region, China. Jiashi County is known as “the hometown of Jiashi muskmelon in China.” It features a warm and dry climate, with an average annual sunshine duration of 2,900 h, average annual temperature of 11.7°C, and annual rainfall of 64.6 mm. In total, 16 rhizosphere soil samples were collected from Jiashi muskmelon greenhouses, including 12 greenhouses replanted for 1, 2, and 3 years (1a, 2a, and 3a, respectively) and 4 greenhouses that had no plantation history (CK). The greenhouses had similar environmental characteristics and were maintained under the same management activities. Basic fertilizer, i.e., farm manure (30,000 kg ha^−1^, organic matter>40%, pH 8.0–9.0) and blended fertilizer (300 kg ha^−1^, N + P_2_O_4_ + K_2_O ≥ 45%), was applied before seeding.

Soil samples were collected on 10 June 2021 (the harvest time of melon in summer). Jiashi muskmelons were pulled from the ground, and rhizosphere soil located within 2 mm of the root surfaces was collected (Zhao et al., 2018). Each treatment was composed of four replicates. Each replicate, consisting of 16 samples, was collected from a different greenhouse using a “Z” pattern. The soil samples were transported on ice to the laboratory. After being screened by a 2-mm sieve, a portion of the soil samples was stored at −80°C for DNA extraction, and the others were air-dried and filtered to determine soil properties.

### Soil physicochemical properties and enzyme activity

The analysis of soil physicochemical properties was based on previous methods (Bao, 2000). Soil pH and electric conductivity (EC) were estimated in a 1:5 (WV^−1^) soil-water suspension. Soil organic matter (SOM) was determined using the potassium dichromate method. Soil available nitrogen (AN) was assessed using the alkaline hydrolysable diffusion method. Available phosphorus (AP) and available potassium (AK) were measured by molybdenum blue colorimetric method and flame photometry. Soil urease (S-UE) activity involves determination of the urea hydrolyzed, and alkaline phosphatase (S-AKP) activity was measured by quantifying the amount of glucose uncombined with orthophosphate ions (Zantua and Bremner, 1975; Buturug et al., 2016). Soil catalase (S-CAT) and sucrase (S-SC) activity was measured by ultraviolet spectrophotometry (Yang et al., 2011) and the 3,5-dinitrosalicylic acid colorimetric method (Parthasarathi and Ranganathan, 2000), respectively.

### Advanced absolute quantification 16S-Seq

Total genomic DNA was extracted from each repeat using the kit (E.Z.N.A. ® Soil DNA Kit, Omega Bio-tek, Norcross, GA, United States). The integrity of genomic DNA was detected through agarose gel electrophoresis, and the concentration was standardized to 10 ng/μL. Thus, in total 16 genomic DNA samples were sent to Genesky Biotechnologies Inc., Shanghai, 201,315 (China) for absolute quantification of 16S rRNA gene amplicon sequencing using Illumina NovaSeq 6000 sequencer. The general description of the absolute quantification 16S-seq were outlined in the study of Tkacz et al. (2018). Briefly, multiple spike-ins with identical conserved regions to natural 16S rRNA genes and variable regions replaced by random sequence with ~40% GC content were artificially synthesized. Then the spike-ins with known gradient spike-in copy numbers were then added to the sample DNA pools. The V4-V5 hypervariable regions of the 16S rRNA gene and spike-ins were amplified with the primer set 515F/907R (Wang W. H. et al., 2020), and bacterial amplicon libraries were produced as described in (Zhou et al., 2022).

### Statistical analysis

The adaptor sequences were removed by the Cutadapt plugin of QIIME2 software. The adaptor and primer sequences were trimmed using the cutadapt plugin. DADA2 plugin was used for quality control and to identify amplified sequence variants (ASVs), which are similar to operational taxonomic units (OTUs). Only sequences >200 bp with an average quality score > 20 and without ambiguous base calls were included. Taxonomic assignments of ASV representative sequences were performed with confidence threshold 0.8 by a pre-trained Naive Bayes classifier which was trained on the RDP (version 11.5). Then the spike-in sequences were identified and reads were counted. A standard curve of read counts versus spike-in DNA copy number was generated, and the absolute copy numbers of bacterial ASVs were counted by using the read counts of the corresponding ASVs. The spike-in sequence was removed prior to subsequent sequence analyzes. The alpha-diversity indices were determined using the vegan package of R software (version R.3.4.3), and the *beta*-diversity indices of the soil bacterial community were calculated using R (version 3.5.1, vegan package). The ASV tables generated were matched to species information in the FAPROTAX database to determine the metabolic functional profiles.

SPSS 20.0 (SPSS Inc., United States) was used for ANOVA statistical analysis, and the drawings were created using R software (version 3.5.1). The Pearson correlation coefficient was used to determine the correlation between the variables. Microbiota composition was assessed by the Bray-Curtis index. The relationship between bacterial communities and environmental factors was determined by redundancy analysis (RDA) using the “vegan” package in R software. The data presented in the study are deposited in the Sequence Read Archive (SRA) repository, accession number PRJNA909159.

## Results

### Soil properties

Environmental factors in rhizosphere soil were affected by the length of continuous cropping ages (Table 1). The concentrations of SOM, AN, and AP increased with the number of replanting years, as did the EC values. However, the AP content first increased and then decreased. After 2 and 3 years of continuous cropping, the soil pH decreased by 9.2 and 9.8%, respectively.

**Table 1:** Rhizosphere soil properties in different samples.

Soil properties	CK	1a	2a	3a
pH	7.41 ± 0.05^a^	6.96 ± 0.04^b^	6.74 ± 0.06^c^	6.69 ± 0.06^d^
EC (μs cm^−1^)	227.45 ± 1.93^d^	617.17 ± 2.08^c^	989.93 ± 1.17^a^	956.27 ± 3.07^b^
SOM (g kg^−1^)	15.07 ± 1.42^d^	17.100 ± 0.44^c^	23.42 ± 0.65^b^	30.02 ± 0.70^a^
AN (mg kg^−1^)	24.50 ± 0.92^d^	35.00 ± 0.7^c^	53.20 ± 0.86^b^	56.00 ± 1.87^a^
AP (mg kg^−1^)	16 ± 0.64^d^	116.79 ± 1.49^c^	178.79 ± 3.8561^b^	264.20 ± 3.0390^a^
AK (mg kg^−1^)	109.09 ± 0.75^d^	231.72 ± 2.13^c^	397.46 ± 1.69^a^	254.50 ± 4.91^b^
S-CAT activity (mg g^−1^ DW)	41.28 ± 0.43^d^	43.93 ± 0.55^bc^	48.62 ± 0.98^b^	50.37 ± 1.84^Aa^
S-UE activity (mg g^−1^ DW)	837.18 ± 1.10^c^	860.71 ± 2.09^b^	919.55 ± 1.86^Aa^	918.69 ± 2.77^a^
S-SC activity (mg g^−1^ DW)	56.38 ± 1.39^d^	111.43 ± 2.78^c^	136.55 ± 3.22^Bb^	916.70 ± 4.15^a^
S-AKP activity (mg g^−1^ DW)	8.40 ± 0.56^d^	10.28 ± 1.09^c^	13.32 ± 0.65^Bb^	15.40 ± 0.76^a^

Values are means ± standard deviation (*n* = 4). EC, electric conductivity; SOM, soil organic matter; AN, available nitrogen; AP, available phosphorus; AK, available potassium; S-CAT, soil catalase; S-UE, soil urease; S-SC, soil sucrase; and S-AKP, alkaline phosphatase. Different letters indicate significant differences (*p* < 0.05). CK, 1a, 2a, and 3a represent greenhouses consecutively planted for 0, 1, 2, and 3 years, respectively.

Based on the soil sample tests, the activities of four enzymes (S-SC, S-UE, S-CAT, and S-AKP) were ranked in the order of CK < 1a <2a < 3a. In a comparison of samples, the enzyme activity values showed significant differences (*p* < 0.05) between each of the enzymes, and the order of the average value variation among the four enzyme activities was S-SC > S-AKP > S-CAT > S-UE.

### Bacterial community phylotypes and diversity

A total of 3,174,891 clean reads were obtained from the 16 samples. The number of identified ASVs per treatment varied from 6,238 to 8,322 and were further classified by the RDP database. A total of 422 ASVs were shared among the four treatments (Supplementary Figure S1), which accounted for 5.2, 5.1, 6.7, and 6.8% of their own ASVs. The unique ASVs accounted for 76.9, 61.4, 53.7, and 59.8%, respectively. The results indicated that microbial species were different in Jiashi muskmelon soil, and continuous cropping promoted the generation of unique species. However, the proportion of unique species decreased over time.

#### Bacterial alpha-diversity

**Figure 1:**
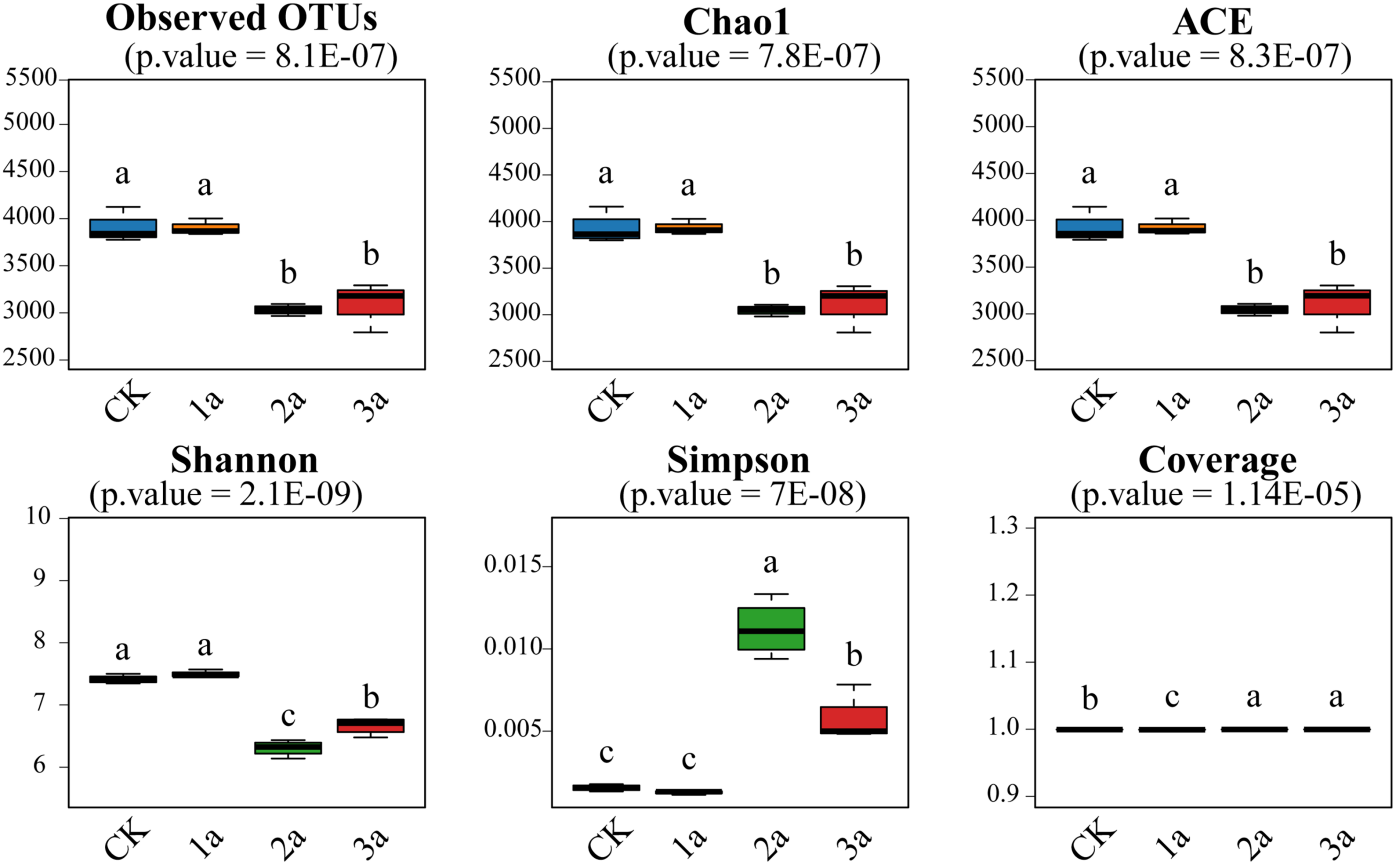
Bacterial alpha diversity of all the tested samples. CK, 1a, 2a, and 3a represent greenhouses consecutively planted for 0, 1, 2, and 3 years, respectively. Different letters indicate significant differences (Tukey’s HSD test, *p* < 0.05; *n* = 4).

The coverage was above 99%, indicating that the sequencing depth was sufficient (Figure 1). The Observed, Chao1, and ACE indices were significantly (*p* < 0.05) decreased with the planting years, but there was no significant difference (*p* > 0.05) during 2 and 3 years of continuous cropping. Although the Shannon index of the 3a samples was higher than that of the 2a samples, it was lower than that of the CK and 1a samples, indicating that continuous cropping reduced bacterial diversity.

#### Bacterial beta-diversity

**Figure 2:**
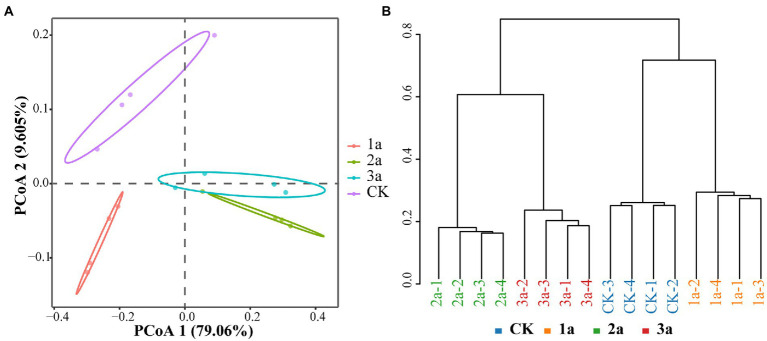
Differences in microbiota community structure based on principal coordinate analysis (**A**, PCoA) and cluster analysis (**B**, calculated on Bray-Curtis). CK, 1a, 2a, and 3a represent greenhouses consecutively planted for 0, 1, 2, and 3 years, respectively.

According to the principal coordinate analysis (PCoA) results, PCoA1 and PCoA2 were attributed to 79.06 and 9.605% of the variation, respectively (Figure 2A), so the correlation was subjected mainly to Axis 1. The 2a and 3a were clustered into one group but separated from CK and 1a by Axis 1, indicating that the bacterial communities were more similar after 2 and 3 years of continuous cropping. This phenomenon was consistent with the Bray-Curtis clustering analysis (Figure 2B). The distribution pattern showed that the bacterial community structure of CK and 1a was significantly different from 2a and 3a, and the bacterial community became more similar with the extension of continuous cropping years.

### Bacterial community composition and structure

**Figure 3:**
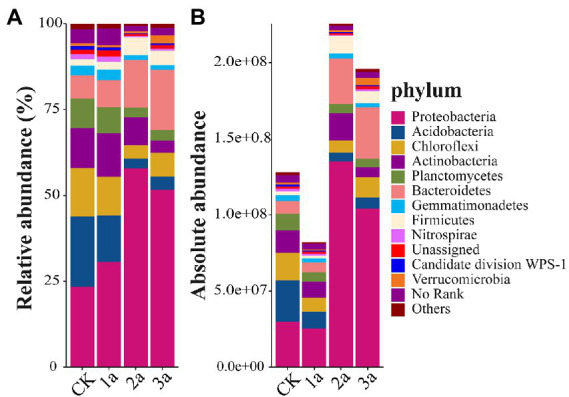
Relative abundances (**A**, %) and absolute abundances (**B**, 16S rRNA gene copies per g of soil) of the main phyla present in all samples. Different bar charts represent different soil samples, and the ordinate is the abundance value. CK, 1a, 2a, and 3a represent greenhouses consecutively planted for 0, 1, 2, and 3 years, respectively.

Quantification of the 16S-seq data was assessed based on the internal spike-in standards and their derived standard curves (e.g., *y* = 0.9541x–0.8684 for the 1a-1 sample, y represents the copy numbers of added spike-in sequences in logarithm, *x* represents the observed ASV reads in logarithm; all fitting coefficients *R*^2^ > 0.99). Estimated absolute copy numbers per g soil at the phylum level were shown in Figure 3B, and the corresponding relative abundances were shown in Figure 3A. The absolute abundance of bacteria after 2 and 3 years of continuous cropping was significantly higher than that of CK. All the tested samples were comprised of 35 phyla. Proteobacteria was the most predominant phylum (relative abundances 23.4% ~ 58.0%), and its absolute abundance increased with continuous cropping duration, especially in 2a and 3a. Acidobacteria was the second most dominant phylum in CK and 1a (relative abundances of 20.6 and 13.5%, respectively), and its absolute abundance decreased continuously. Bacteroidetes was the second most dominant phylum in 2a and 3a (relative abundances of 13.8 and 17.6%, respectively), and its absolute abundance began to increase after 2 years of continuous cropping. The absolute abundances of Chlorobacteria and Nitrospirae initially decreased and then increased but were still lower in continuous cropping soil than in CK. The absolute abundances of Actinobacteria and Planctomycetes were significantly lower after long-term continuous cropping. The absolute abundance of Firmicutes increased after 2 years of continuous cropping; in contrast, the absolute abundance of Gemmatimonadetes dropped. These results showed that bacterial community members at the phylum level were regulated by prolonged greenhouse cultivation, and the longer the replanting years were, the more obvious the changes in bacterial community and abundance.

**Figure 4:**
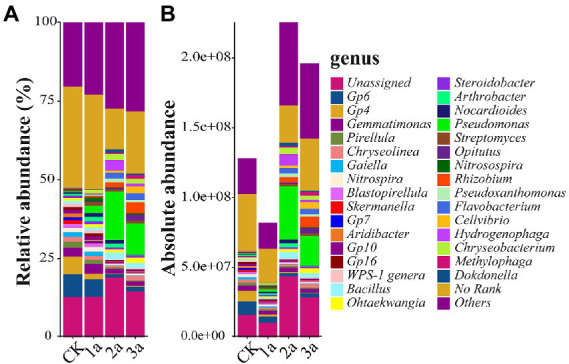
Relative abundances (**A**, %) and absolute abundances (**B**, 16S rRNA gene copies per g of soil) of the main genus present in all samples. CK, 1a, 2a, and 3a represent greenhouses consecutively planted for 0, 1, 2, and 3 years, respectively.

The top 50 abundant bacterial genera accounted for more than 65% of the total abundance, and all of these genera exhibited significant differences in average absolute abundance between treatments. *Gemmatimonas* was the common dominant genus (>2.5 × 10^7^ copy/g soil in at least one soil sample; Figure 4), whereas *Pseudomonas* was the primary genus. Others, such as *Hydrogenophaga*, *Bacillus*, *Chryseobacterium*, *Flavobacterium*, *Rhizobium*, *Arthrobacter*, *Pseudoxanthomonas*, and *Nocardioides*, were the dominant genera in one or two samples. Of the top 10 abundant bacterial genera, the absolute abundances of *Hydrogenophaga*, *Bacillus*, and *Chryseobacterium* increased continuously within 2 years of continuous cropping and then dropped but were still much higher than the absolute abundance of CK. The absolute abundance of *Flavobacterium* and *Rhizobium* increased continuously as the number of planted years increased.

#### LEfSe analysis

**Figure 5:**
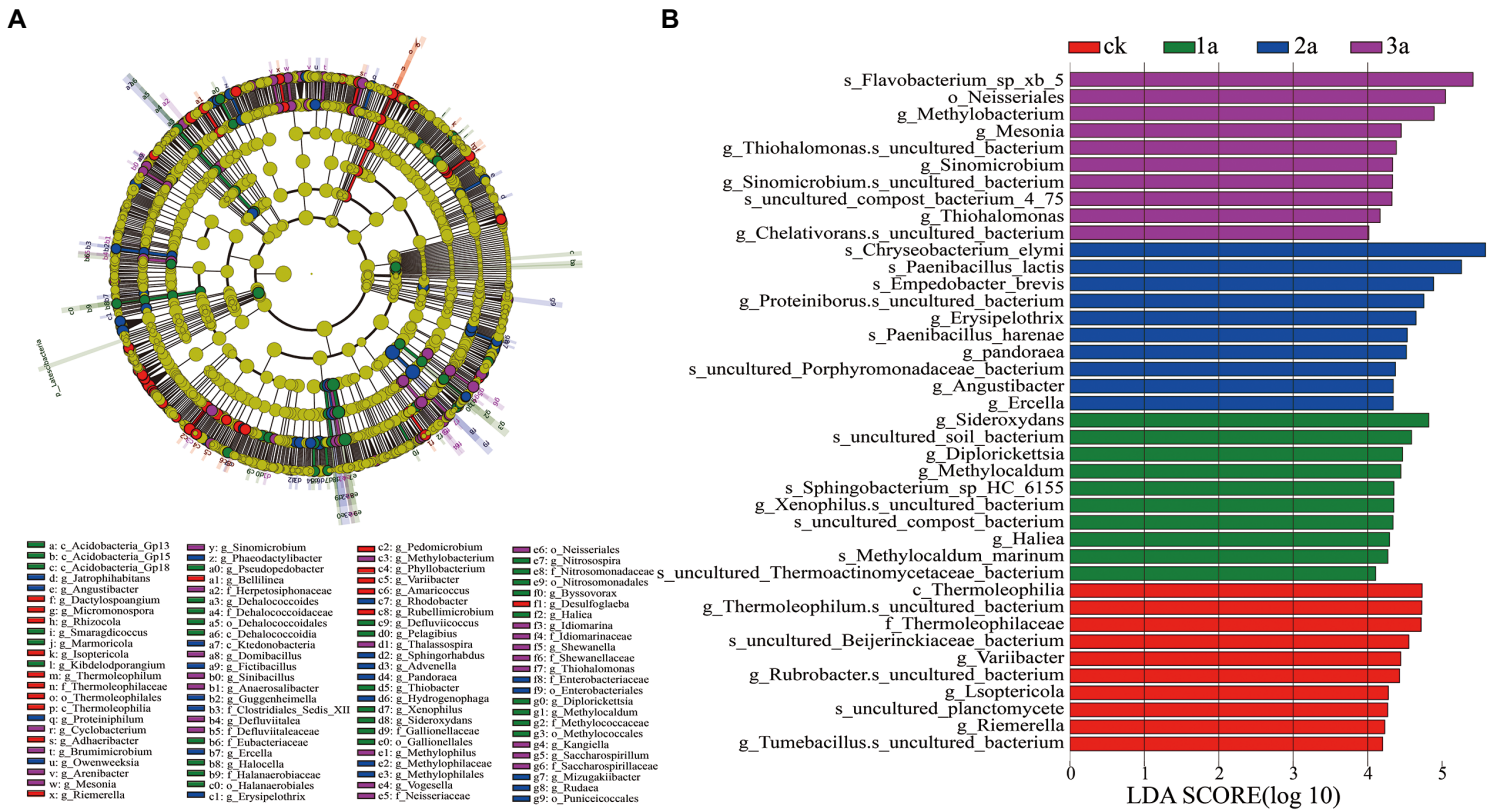
Evolutionary branch diagram of bacterial community differences **(A)**. Indicator microbes in groups with linear discriminant analysis (LDA) scores greater than 4 **(B)**. CK, 1a, 2a, and 3a represent greenhouses consecutively planted for 0, 1, 2, and 3 years, respectively. Each circle represents all taxonomic units at a taxonomic level, and the circle from inside to outside represents the phylogenetic level from the phylum to genus or species. Nodes on the circle represent taxa at the taxonomic level, and the diameter of each circle is proportional to the absolute abundance of bacteria.

Based on LDA (Linear Discriminant Analysis) effect size (LEfSe) analysis, the abundances of microbial species in Jiashi muskmelon continuous cropping soil were significantly different (Figure 5). A total of 40 biomarkers with LDA scores >4 were enriched across the samples. Of the 10 taxa in CK, the largest contribution was from *Thermoleophilum* (at the genus level) and Thermoleophilaceae (at the family level). There were 10 taxa in 1a, and the most important contribution was *sideroxydans* (at the genus level), which can drive the iron cycle in soil. Ten taxa were observed in 2a, and the largest contributions were made by the drug-resistant strain *Chryseobacterium elymi* and the biocontrol strain *Paenibacillus lactis* (at the species level). Ten taxa were observed in 3a, and the largest contributor was the opportunistic pathogen *Flavobacterium*. Xb5 (at the species level), Neisseriales (at the order level), and *Methylobacterium* and *Mesonia* (at the genus level).

### Functional prediction analysis

**Figure 6:**
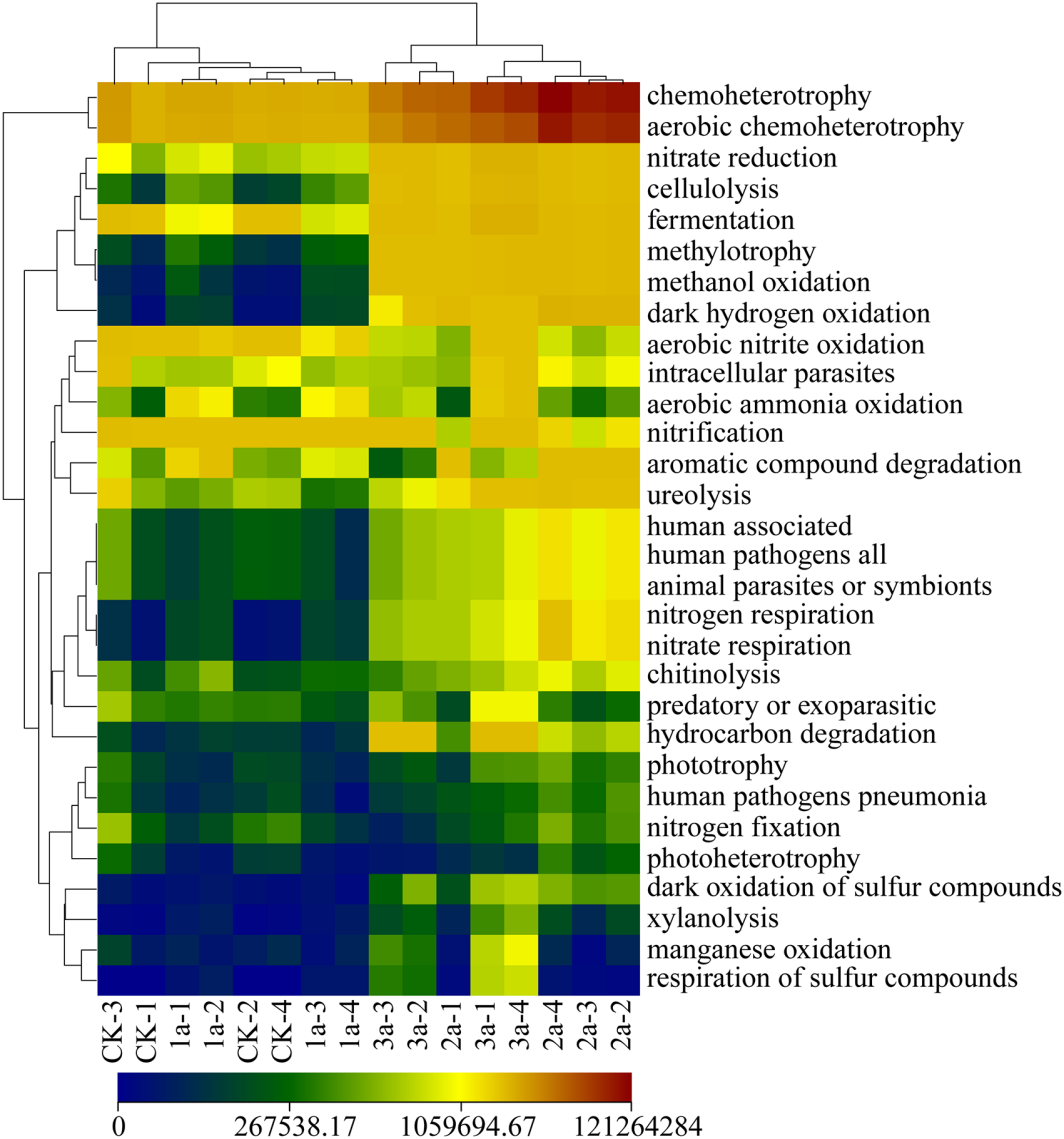
Heatmap of microbial community function in the FAPROTAX database among the 16 samples. CK, 1a, 2a, and 3a represent greenhouses consecutively planted for 0, 1, 2, and 3 years, respectively.

A total of 29 functional groups were predicted in soil based on the ASVs (Figure 6). There were 6, 6, 15, and 13 taxa with absolute abundance >10^6^ copy/g soil in all the tested samples, among which 9 taxa were shared in 2a and 3a, while only 4 taxa were shared in CK and 1a. The taxa associated with chemoheterotrophy and aerobic chemoheterotrophy were predominant, followed by fermentation and nitrate reduction. The absolute abundance of the taxa associated with nitrate reduction and cellulolysis showed a continuous upward trend, while that of chemoheterotrophy, aerobic chemoheterotrophy, and fermentation increased significantly after long-term continuous cropping. Cluster analysis also showed that functional groups were clustered according to continuous cropping years, that is, 1a and CK were grouped into a cluster, while 2a and 3a were grouped into another cluster. Hence, continuous cropping changed microbial function, while long-term continuous cropping was more likely to form common groups.

### Relationships between bacterial community and soil properties

Spearman’s rank-order correlation indicated that the absolute abundances of Proteobacteria, Bacteroidetes, Firmicutes, and Verrucomicrobia were negatively correlated with pH and positively correlated with the other factors (Table 2). The absolute abundances of Acidobacteria, Chloroflexi, Planctomycetes, and Gemmatimonadetes were positively correlated with pH and negatively correlated with the other factors. The absolute abundances of Actinobacteria were positively correlated with pH and EC and negatively correlated with the other factors.

**Table 2:** Spearman’s rank correlations between abundant bacterial phyla/genera and soil properties.

Phyla/genera	pH	EC	SOM	AN	AP	AK	S-CAT	S-UE	S-SC	S-AKP
Proteobacteria	−0.811^**^	0.835^**^	0.665^**^	0.665^**^	0.665^**^	0.806^**^	0.665^**^	0.765^**^	0.697^**^	0.662^**^
Bacteroidetes	−0.637^**^	0.635^**^	0.718^**^	0.741^**^	0.738^**^	0.656^**^	0.656^**^	0.726^**^	0.741^**^	0.727
Acidobacteria	0.887^**^	−0.871^**^	−0.788^**^	−0.765^**^	−0.765^**^	−0.876^**^	−0.824^**^	−0.776^**^	−0.756^**^	−0.786^**^
Actinobacteria	0.019	0.026	−0.485	−0.491	−0.479	−0.041	−0.447	−0.215	−0.438	−0.487
Chloroflexi	0.558^*^	−0.544^*^	−0.300^*^	−0.262	−0.285	−0.529^*^	−0.409	−0.303	−0.282	−0.330
Planctomycetes	0.477	−0.453	−0.424	−0.388	−0.400	−0.429	−0.500	−0.341	−0.391	−0.424
Firmicutes	−0.740^**^	0.738^**^	0.626^**^	0.638^**^	0.638^**^	0.759^**^	0.591^**^	0.721^**^	0.644^**^	0.633^**^
Gemmatimonadetes	0.085	−0.044	−0.221	−0.200	−0.194	−0.062	−0.274	−0.021	−0.156	−0.221
Verrucomicrobia	−0.309	0.315	0.568^*^	0.582^*^	0.597^*^	0.318	0.553	0.506	0.624	0.586
Nitrospirae	0.806^**^	−0.794^**^	−0.547^*^	−0.497^*^	−0.512^*^	−0.782^**^	−0.629^*^	−0.565^*^	−0.503^*^	−0.528^*^
*Pseudomonas*	−0.905^**^	0.903^**^	0.785^**^	0.797^**^	0.797^**^	0.918^**^	0.732^**^	0.879^**^	0.797^**^	0.786^**^
Gp6	0.867^**^	−0.829^**^	−0.744^**^	−0.709^**^	−0.712^**^	−0.853^**^	−0.762^**^	−0.729^**^	−0.700^**^	−0.728^**^
*Gemmatimonas*	0.085	−0.044	−0.221	−0.200	−0.194	−0.062	−0.274	−0.021	−0.156	−0.221
*Rhizobium*	−0.730^**^	0.735^**^	0.853^**^	0.847^**^	0.853^**^	0.715^**^	0.800^**^	0.826^**^	0.874^**^	0.829^**^
*Hydrogenophaga*	−0.943^**^	0.932^**^	0.756^**^	0.779^**^	0.785^**^	0.953^**^	0.724^**^	0.885^**^	0.782^**^	0.761^**^
Gp4	0.840^**^	−0.844^**^	−0.826^**^	−0.821^**^	−0.844^**^	−0.850^**^	−0.921^**^	−0.779^**^	−0.847^**^	−0.855^**^
*Bacillus*	−0.714^**^	0.712^**^	0.600^*^	0.612^*^	0.615^*^	0.732^**^	0.571^*^	0.697^**^	0.612^**^	0.600^**^
*Chryseobacterium*	−0.896	0.891	0.803	0.806	0.812	0.909	0.741	0.874	0.800	0.792
*Flavobacterium*	−0.802^**^	0.812^**^	0.856^**^	0.876^**^	0.879^**^	0.818^**^	0.788^**^	0.897^**^	0.876^**^	0.851^**^
*Ohtaekwangia*	−0.590^*^	0.585^*^	0.659^**^	0.694^**^	0.694^**^	0.609^*^	0.621^*^	0.697^**^	0.718^**^	0.687^**^
*Chryseolinea*	0.181^**^	−0.174^**^	0.185^**^	0.232^**^	0.235^**^	−0.156^**^	0.156^**^	0.103^**^	0.265^**^	0.231^**^
*Cellvibrio*	−0.746^**^	0.724^**^	0.897^**^	0.903^**^	0.900^**^	0.729^**^	0.850^**^	0.853^**^	0.938^**^	0.890^**^
*Arthrobacter*	−0.356	0.371	−0.224	−0.226	−0.229	0.341	−0.206	0.088	−0.203	−0.237
*Opitutus*	−0.593^*^	0.624^*^	0.791^**^	0.782^**^	0.791^**^	0.588^**^	0.785^**^	0.732^**^	0.832^**^	0.806^**^
*Pirellula*	0.664^**^	−0.644^**^	−0.724^**^	−0.712^**^	−0.744^**^	−0.659^**^	−0.794^**^	−0.629^**^	−0.741^**^	−0.752^**^
*Nitrospira*	0.806^**^	−0.794^**^	−0.547^*^	−0.497^*^	−0.512^*^	−0.782^**^	−0.629^**^	−0.565^*^	−0.503^*^	−0.528^*^

EC, electric conductivity; SOM, soil organic matter; AN, available nitrogen; AP, available phosphorus; AK, available potassium; S-CAT, soil catalase; S-UE, soil urease; S-SC, soil sucrase; and S-AKP, alkaline phosphatase. *p < 0.05, **p < 0.01.

**Figure 7:**
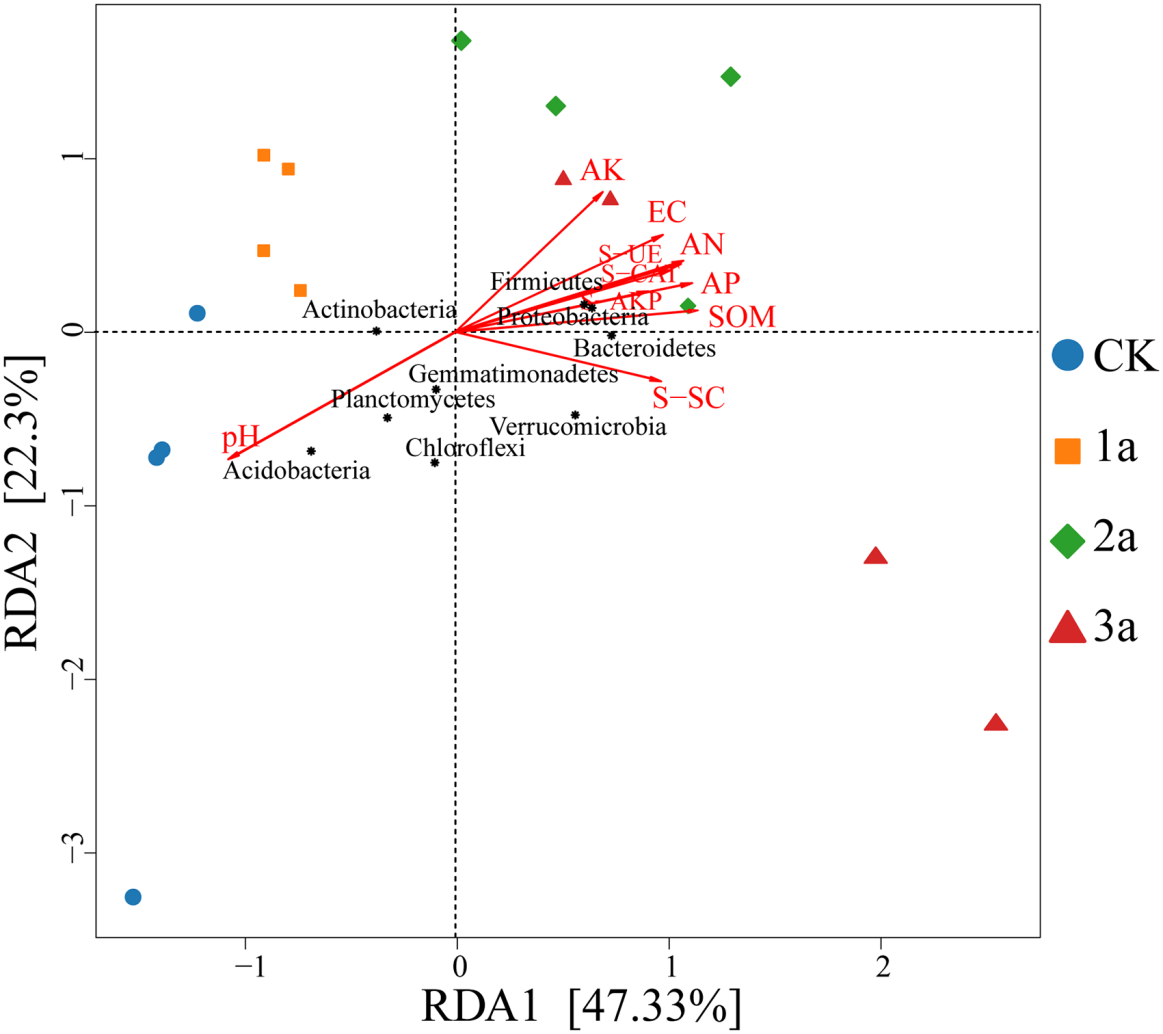
Redundancy analysis (RDA) identified the correlation between environmental variables and dominant bacterial plyla. CK, 1a, 2a, and 3a represent greenhouses consecutively planted for 0, 1, 2, and 3 years, respectively. EC, electric conductivity; SOM, soil organic matter; AN, available nitrogen; AP, available phosphorus; AK, available potassium; S-CAT, soil catalase; S-UE, soil urease; S-SC, soil sucrase; and S-AKP, alkaline phosphatase.

RDA analysis was conducted to identify the impact of soil properties on abundant phyla (Figure 7). The first axis explained 47.33% of the variance, while the second axis only explained 22.3%. The structure of bacterial communities from the second and third years were related to low pH in contrast to CK and the first year, while the other factors were the opposite. Overall, the environmental variables that had a significant influence on bacterial community structure were AN (*r*^2^ = 0.913, *p* = 0.001), S-AKP (*r*^2^ = 0.897, *p* = 0.001), EC (*r*^2^ = 0.888, *p* = 0.001), and S-UE (*r*^2^ = 0.885, *p* = 0.001).

## Discussion

The richness and diversity of soil microorganisms can comprehensively reflect the overall changes in microbial flora, which indicates the health status of soil. The bacterial richness and diversity decreased in Jiashi muskmelon continuous cropping soil, although there were more soil nutrients, which was consistent with previous studies (Nayyar et al., 2009; Liu N. et al., 2020). In addition, the number of microbial species decreased, indicating that only a few populations could adapt to changes in the soil environment. Our analysis of recent studies shows that different microbial species have different variation trends under the influence of various factors, such as crop type, continuous cropping years, and daily management, but the ratio of bacteria to fungi in soil showed a decreasing trend. The transformation of the “fungal type” was obvious, leading to the deterioration of the soil micro ecological environment (Li et al., 2017). Generally, the richer the soil microbial diversity is, the better the stability of the microbial community and the better the resistance to environmental disturbances. Thus, the loss of rhizosphere bacterial richness and diversity may be one of the factors that resulted in the poor growth of melon plants.

The most dominant bacterial phyla were Proteobacteria and Acidobacteria, consistent with previous studies (Li et al., 2016). Proteobacteria belong to the eutrophic phyla associated with long periods of intense production (Dai et al., 2018), which may be consistent with the increased abundance in our study. Acidobacteria is identified as an oligotrophic (or K-selected) group in soil that is easily enriched in low-fertility soils (Ai et al., 2018). The responses of the two phyla to continuous cropping measures were consistent with their nutrient classification. However, the absolute abundance of the eutrophic phylum Actinomycetes decreased, while the absolute abundance of the oligotrophic phylum Firmicutes increased, indicating that the continuous cropping regime changed their growth strategy. Proteobacteria is one of the fastest metabolizing bacteria that plays a crucial role in maintaining soil ecological stability by soil nitrogen supply (Ren et al., 2020). However, the enrichment of Bacteroidetes is unfavorable to plants, as members of them are plant pathogenic bacteria. Actinobacteria can produce antibiotics to inhibit plant pathogens and decompose soil organic matter (Zhang et al., 2017). Its abundance was positively correlated with soil pH in our study, inconsistent with the negative correlation between soil Actinobacteria abundance and soil pH reported in previous reports (Haichar et al., 2014). Gemmatimonadetes was reported to be particularly affected by soil physicochemical properties and positively related to soil moisture content (Ren et al., 2020). Thus, the continuous cropping of melon may lead to decreased water-holding capacity, which needs to be further studied. Nitrospirae is closely related to soil nitrite oxidation, which affects crop N uptake (Daims et al., 2016). However, it is more suitable for a high pH environment, and acidic soils could inhibit their activity. Similar results were also observed in potato, *Rehmannia glutinosa*, and coffee (Liu et al., 2014; Wu et al., 2018; Zhao et al., 2018). Firmicutes can produce spores to resist external harmful factors with strong resistance. Its increased abundance may improve the ability of soil to resist disease risk. Manure was reported to be enriched in Firmicutes (Tu et al., 2018), and the habit of application of organic manure may lead to the enrichment of Firmicutes members. These differences in different treatments further confirm that the abundance and structure of soil bacterial communities are influenced by continuous cropping (Li et al., 2019; Liu Z. X. et al., 2020).

At the genus level, *Pseudomonas* could strengthen the cell wall of roots against pathogens by inducing changes in plant cell wall structure (Benhamou, 1996). *Hydrogenophaga* and *Chryseobacterium* are capable of reducing toxic substances in soil, such as PAHs and phenols. Many species of *Bacillus*, which are recognized as environmentally friendly soil regulators, can produce effective substances to control soil-borne pathogens (Shen et al., 2018). *Rhizobium* can invade plant root hairs to form nodules and then differentiate into branched polymorphic cells for symbiosis with plants to fix nitrogen. The increased abundance of these genera suggested that they may play important roles in melon continuous cropping soil. *Flavobacterium* is an opportunistic pathogen that exists widely in the environment, and its increased density will interfere with the healthy soil microenvironment. *Gemmatimonas* is involved in phosphonate and phosphinate metabolism (Xun et al., 2021). *Arthrobacter* strains have strong environmental adaptability and stress resistance and can degrade various kinds of environmental organic pollutants and adsorb heavy metal ions (Tarfeen et al., 2022). In addition, *Arthrobacter* can degrade nicotine and resist insect attack (Brown, 1974). *Nocardioides* is a Gram-positive bacterium that is distributed mainly in soil. Some *Nocardioides* strains are also plant growth-promoting bacteria (PGPB) capable of secreting antibiotics, producing siderophores and 1-aminocyclopropane-1-carboxylate (ACC) deaminase (Gong et al., 2018). The decrease of these genera may lead to negative impacts on disease resistance during continuous cropping.

Chemoheterotrophy and aerobic chemoheterotrophy were the most predominant groups, which concurred with previous findings (Liang S. C. et al., 2020). They are also universal ecosystem functions performed by most microbes, such as Acidobacteria and Proteobacteria (Yan et al., 2018). The increased abundances of chemoheterotrophy and aerobic chemoheterotrophy were probably due to the increase in soil nutrients. In addition, they may also be affected by root exudates. Continuous cropping was reported to be able to enrich the soil in root exudates, such as sugar and amino acids (Strickland et al., 2015; Wu et al., 2019). Then, their accumulation will provide more carbon, nitrogen, and energy sources for chemoheterotrophic microorganisms (Bais et al., 2006). Chemoheterotrophy, aerobic chemoheterotrophy, and fermentation are important ecological functions related to the carbon cycle (Liang Z. S. et al., 2020). Their increased abundances indicate that a higher proportion of bacteria were involved in carbon cycling under the continuous cropping system, which will promote the mineralization of soil organic carbon. Root exudates can stimulate the metabolic activity of denitrifying bacteria (Giles et al., 2017; Langarica-Fuentes et al., 2018). However, nitrate reduction would cause the loss of nitrogen fertilizer in the soil, implying potential nutrient loss in melon continuous cropping soil.

In intensive agricultural systems, increasing nitrogen fertilizer is the main management strategy to obtain a high yield. However, the abundance of pathogenicity-related genes increased under high nitrogen stress (Wei et al., 2018), so soil pathogens will secrete more disease-causing genes or enzymes to extract nutrients from the environment. The AP content decreased over time, which was not conducive to the excellent quality of the melon. In addition to the unique light and water conditions in Jiashi County, the most important reason for the excellent character of Jiashi muskmelon is that the soil here is rich in AP, with an average content of more than 210 ppm. Hence, the application of K fertilizer should be increased during melon cultivation.

Due to anthropogenic impacts such as excessive fertilization and unreasonable disturbance, the soil in greenhouses rapidly acidifies in 10–20 years or even less (He et al., 2019; Arafat et al., 2020; Na et al., 2021). Melons will grow better in neutral soil, while acidic environment after continuous cropping may limit root development and nutrient uptake in the plant growth process. Furthermore, it will promote the proliferation of soil-borne pathogens (Sun et al., 2014) and increase the incidence of soil-borne pathogens (Kim et al., 2016). Thus, for melon cultivation, alkaline materials such as lime and biochar can be used to improve the acidic environment and balance soil nutrients, especially secondary microelements (Gao et al., 2019; Li et al., 2022). The increased soil EC value indicates that soil salinization is aggravated, which may be an important factor for the poor growth of greenhouse melon.

To date, the mitigation measures for continuous cropping soil diseases remain controversial. There is no set of perfect soil management measures that could coordinate all factors, so the rhizosphere micro ecosystem will be repeatedly managed but repeatedly unbalanced. Since the dynamic change in root exudate composition directly influences the assemblage and activities of rhizosphere microbiota (Jin et al., 2020; Vives-Peris et al., 2020; Zhang et al., 2020), the complex processes and molecular mechanisms of root exudation-soil-microbial interactions need to be further studied to establish a sound rhizosphere micro ecosystem theory. Meanwhile, the application of microbial methods is essential, such as the development of special microbial fertilizer for melon to construct an immune soil environment.

## Conclusion

In summary, continuous cropping in Jiashi muskmelon cultivation led to significant changes in soil properties. Under continuous cropping conditions, the total abundance of rhizosphere bacteria increased, but the diversity and richness decreased significantly. Furthermore, bacterial community structure and function were also significantly altered, and the most dominant phyla were Proteobacteria and Actinobacteria. The reduction in diversity and abundance of soil microbes could be the main factors for the reduced yield and quality and soil-borne diseases in Jiashi muskmelon. The abundances of bacterial groups capable of chemoheterotrophy, aerobic chemoheterotrophy, fermentation, and nitrate reduction increased over time. The results provide helpful information on the differences in soil microbial communities in continuous cropping systems of melon. During actual melon production, attention should be given to the application of microbial methods such as exploring soil amendments and microbial fertilizers to alleviate continuous cropping soil sickness.

## Data availability statement

The datasets presented in this study can be found in online repositories. The names of the repository/repositories and accession number(s) can be found in the article/Supplementary material.

## Author contributions

JW and ML designed the experiment and edited the manuscript. QZ carried out the field sampling. TZ took part in data analysis. All authors contributed to the article and approved the submitted version.

## Funding

This work was supported by Xinjiang Uygur Autonomous Region University Scientific Research Project (XJEDU2021Y039) and the National Nature Science Foundation of China (32160408).

## Conflict of interest

The authors declare that the research was conducted in the absence of any commercial or financial relationships that could be construed as a potential conflict of interest.

## Publisher’s note

All claims expressed in this article are solely those of the authors and do not necessarily represent those of their affiliated organizations, or those of the publisher, the editors and the reviewers. Any product that may be evaluated in this article, or claim that may be made by its manufacturer, is not guaranteed or endorsed by the publisher.

## Supplementary material

SUPPLEMENTARY FIGURE S1 Amplified sequence variants (ASVs) -based petal maps. CK, 1a, 2a, and 3a represent greenhouses consecutively planted for 0, 1, 2, and 3 years, respectively.
