# Supplementary figures and images for: Effects of continuous cropping Jiashi muskmelon on rhizosphere microbial community
Source: Front Microbiol. 2023 Jan 9;13:1086334. doi: 10.3389/fmicb.2022.1086334 (PMC9868712; doi:10.3389/fmicb.2022.1086334)

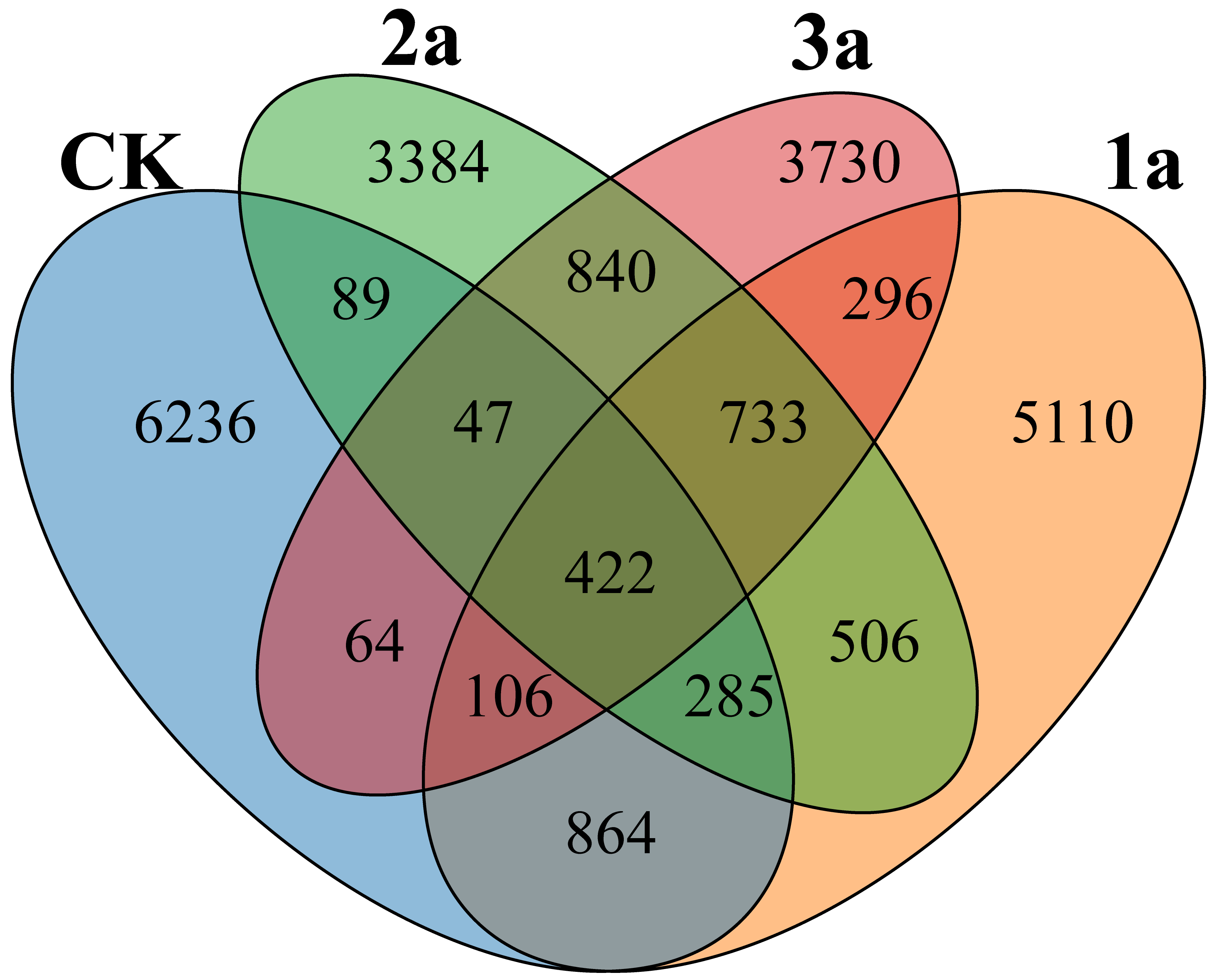

Supplement: SUPPLEMENTARY FIGURE S1 — Amplified sequence variants (ASVs) -based petal maps. CK, 1a, 2a, and 3a represent greenhouses consecutively planted for 0, 1, 2, and 3 years, respectively. [file Image_1.TIF]
